# Data Collection Variability Across Neonatal Hypoxic-Ischemic Encephalopathy Registries

**DOI:** 10.1016/j.jpeds.2025.114476

**Published:** 2025-01-23

**Authors:** Eric S. Peeples, Ulrike Mietzsch, Eleanor Molloy, Gabrielle deVeber, Khorshid Mohammad, Janet S. Soul, Danielle Guez-Barber, Betsy Pilon, Vann Chau, Sonia Bonifacio, Jehier Afifi, Alexa Craig, Pia Wintermark

**Affiliations:** 1 Department of Pediatrics, University of Nebraska Medical Center, Omaha, NE; 2 Division of Neonatology, Children’s Nebraska, Omaha, NE; 3 Child Health Research Institute, Omaha, NE; 4 Department of Pediatrics, University of Washington School of Medicine, Seattle, WA; 5 Division of Neonatology, Seattle Children’s Hospital, Seattle, WA; 6 Department of Pediatrics, Trinity College Dublin, Dublin, Ireland; 7 Children’s Hospital Ireland, Dublin, Ireland; 8 The Hospital for Sick Children, Toronto, Ontario, Canada; 9 Department of Pediatrics, University of Toronto, Toronto, Ontario, Canada; 10 Department of Pediatrics, University of Calgary, Calgary, Alberta, Canada; 11 Division of Pediatric Neurology, Boston Children’s Hospital, Boston, MA; 12 Department of Pediatrics, Harvard Medical School, Cambridge, MA; 13 Children’s Hospital Colorado, Aurora, CO; 14 Department of Pediatrics, University of Colorado, Aurora, CO; 15 Hope for HIE, West Bloomfield Township, MI; 16 Lucile Packard Children’s Hospital, Palo Alto, CA; 17 Department of Pediatrics, Stanford University School of Medicine, Stanford, CA; 18 Department of Pediatrics, Dalhousie University, Halifax, Nova Scotia, Canada; 19 Department of Pediatrics, Barbara Bush Children’s Hospital at Maine Health, Portland, ME; 20 Department of Pediatrics, Tufts University School of Medicine, Boston, MA; 21 Department of Pediatrics, McGill University, Montreal, Quebec, Canada; 22 Montreal Children’s Hospital, Montreal, Quebec, Canada

## Abstract

**Objective:**

To assess variability among data elements collected among existing neonatal hypoxic-ischemic encephalopathy (HIE) data registries worldwide and to determine the need for future harmonization of standard common data elements.

**Study design:**

This was a cross-sectional study of data elements collected from current or recently employed HIE registry data forms. Registries were identified by literature search and email inquiries to investigators worldwide. Data elements were categorized by group consensus.

**Results:**

A total of 1281 data elements were abstracted from 22 registries based in 14 countries, including 3 middle-income countries. Registries had a median of 106.5 distinct data elements per registry (range 59–458). The most commonly collected data were related to pregnancy, therapeutic hypothermia, and short-term hospital outcomes. The least consistently collected data were laboratory values other than acid/base status values. Only 4 variables were consistently collected in every registry. Five registries included neurodevelopmental follow-up fields and 5 others linked their data to a separate follow-up registry.

**Conclusion:**

Many HIE registries are collecting patient data around the world, but there is considerable variability in the number, type, and format of data collected. Future attempts to develop standard common data elements to harmonize data collection globally will be crucial to facilitate worldwide collaboration and to optimize management and outcome of neonatal HIE.

Hypoxic-ischemic encephalopathy (HIE) – a cause of neonatal encephalopathy (NE) and a potential result of perinatal asphyxia – is a serious neonatal condition with an incidence of 1–4 per 1000 live births in high-income countries (HICs)^1,2^ and up to 31 per 1000 live births in low- and middle-income countries (LMICs).^2,3^ Perinatal management of neonates with HIE is complex as HIE is frequently accompanied by multiorgan failure, and despite current care algorithms, many infants die or survive with significant morbidities.^4^ Clinicians seeking to improve care for this vulnerable population face several challenges, including wide variability in management and the lack of robust evidence for many aspects of care.^5^

As therapeutic hypothermia (TH) became standard of care in HICs over the past decade, local, regional, and national data registries emerged with the purpose of collecting longitudinal surveillance data – including information about treatment, outcomes, and practice changes – and supporting quality improvement and research.^6–12^ Registries can also provide observational research data when large randomized trials are not feasible.^13^ However, there is variability in the recording of practices and outcomes at each of those levels (local, regional, national and international).^14–16^ In addition, given the cost and time burden of developing and maintaining a registry, individual registries are limited in the number of centers and neonates included, further limiting comparison of practices and outcomes among centers.

The largest HIE registries were initiated as part of TH clinical trials or as part of for-fee registries such as the Vermont Oxford Network, Canadian Neonatal Network, or Children’s Hospital National Consortium database.^17–19^ These for-fee registries often involve large consortia and therefore many patients, but they collect data on many neonatal diagnoses, so they are restricted in the amount of HIE-focused data that can be collected. Conversely, some registries are highly selective studying only neonates with HIE. Although they tend to have more disease-specific data, they are typically much smaller than the general registries due to their site- or region-specific nature and may not reflect the entire scope of the population of neonates with HIE. Regardless of the intent of the registry, however, if data were harmonized, existing registries would be able to pool data, greatly increasing sample size. This could lead to harmonized benchmarking, significant improvement in the understanding of this complex disease, and optimization of treatment approaches and examination of previously unrecognized research questions.

As a low-incidence and high-risk condition, the care of neonates with HIE will benefit from data harmonization across studies and registries. As a first step toward harmonization of data, we sought to survey current neonatal HIE/NE data registries to assess similarities and discrepancies among the included data elements and format of the variables collected.

## Methods

We conducted a worldwide cross-sectional study examining the prevalence of data elements from any existing or recently used local, regional, national, or international registries that include data collection on neonates with HIE, NE, and/or perinatal asphyxia. Registries were identified by literature search and email inquiries to investigators. We included only contemporary registries that were developed after the adoption of TH. Data entry forms of participating registries were collected, and individual data elements were extracted. In addition, information on data entry format was collected (eg, free text, categorical, binary yes/no answers, multiple-choice answers, numerical answers with units of measure, date/time fields, etc.). For multiple-choice answers, the options for each variable were noted.

Data elements were grouped into the following categories as determined by study group consensus: sociodemographics, prenatal/pregnancy, labor and delivery including sentinel events, neonatal resuscitation and delivery room events, acid-base status, other laboratory values, and information from the neonatal course including transport, TH, neurological examination, neuromonitoring and seizures, and neuroimaging findings. Additionally, the overall hospital course and short-term outcomes were subdivided into subcategories of neurological, respiratory, cardiovascular, renal and fluids, feeding and nutrition, gastrointestinal (including hepatic), infectious, and condition at discharge.

We deployed a RedCap^20^ survey from October 2023 through January 2024 to the lead investigators of the included registries to collect further details regarding their registry, including the terminology used (ie, HIE, NE, and/or PA), the primary purpose(s) of their registry, the inclusion/exclusion criteria, the geographic area covered by the registry (local, regional national, international), the number of hospitals participating in the registry, and the level of care provided by each site of their registry (TH, noninvasive positive pressure ventilation, invasive mechanical ventilation, high-frequency ventilation, extracorporeal membrane oxygenation, and surgical management). The registries were also surveyed for the presence or absence of follow-up data or linkage to follow-up registries, the maximum age to which follow-up is provided, the registry funding source(s), the use and type of electronic medical record system(s), and the interest and ability to harmonize common data elements of their existing registry and participate in a larger international registry. The full survey is displayed as Supplemental File 1; available at www.jpeds.com. In cases where the included registry was no longer active and no representative was reachable to complete the survey, survey answers were abstracted from published literature as feasible.

Data were analyzed with Stata version 14.2 (StataCorp), using descriptive statistics to summarize and characterize variability in data elements between registries.

## Results

Worldwide, 26 registries were identified, and 22 registries chose to contribute their data element lists to our study (Supplemental File 2; available at www.jpeds.com). One registry chose not to participate, 2 could not be reached despite multiple attempts, and one contributed a coded data element list but did not provide a key to the element codes. The participating registries represented 14 countries, including 11 HICs and 3 middle-income countries (Table I). Although requests were made to other potential LMIC participants, there was either a lack of response or lack of any existing registry near the contact, typically related to low resources available for these types of registries.

After compiling data element lists from all registries, 1281 data elements were identified (Supplemental Table 1; available at www.jpeds.com provides a complete list of data elements). After collapsing elements that could be easily derived from other elements (ie, the binary variable of whether positive pressure was administered in the delivery room vs the continuous variable of how long positive pressure was administered in the delivery room), 1074 distinct elements remained. There was a median of 106.5 data elements per registry (range 59–458). Table II demonstrates the element stratification by category. The most commonly collected data elements were related to pregnancy, hypothermia therapy, and short-term hospital outcomes. In 5 registries (23%), no neuroimaging data were collected. Two of these registries were among the 3 from middle-income countries. The least consistently collected data were laboratory values not related to acid/base status.

Only 4 variables were collected in all 22 registries. The top 22 data elements – representing elements reported by 13 or more registries – are shown in Table III. Even when elements were collected by multiple registries, the format of the individual data element (numeric, categorical, free text, etc.) often differed across registries. Most registries (86.4%) included at least one free text response element, with a median of 3 free text response elements (range 0–19) per registry. Five registries (22.7%) included fields reporting developmental outcome at follow-up and 5 others (22.7%) linked their data to a separate follow-up registry. Follow-up data were collected to a median age of 4 years (range 2–8 years).

Responses to the follow-up survey were collected from 19/22 (86%) participating registries. Most of the registries (11/19, 58%) used the term “hypoxic-ischemic encephalopathy” to define their registry sample, with others using the terms “asphyxia” (2/19, 10%) or “neonatal encephalopathy.” (3/19, 16%) Three registries did not use these terms, preferring to describe their sample as “therapeutic hypothermia.” (3/19, 16%) Despite the differences in terminology, all registries enrolled subjects with neonatal encephalopathy who underwent TH. The different registry study samples and terms and the associated inclusion and exclusion criteria are listed in Supplemental Table 2; available at www.jpeds.com. Most of the registries were developed for the purpose of local quality improvement initiatives (16/19, 84%) and/or prospective or retrospective research (12/17, 71%, 2 did not respond). The funding sources for the registries varied widely, from local or national grant funding (5/19, 26%) to foundation funding (5/19, 26%), state or national governmental funds (4/19, 21%), fees collected from participating sites (2/19, 10%), or insurance companies (2/19, 10%). Four registries (21%) reported no funding. Of those who completed the full survey, 12 of 13 (92%) stated that they would be interested in attempting to harmonize data between studies. The one site that responded that they would not be interested did not provide any comments explaining potential barriers or hesitance to harmonization.

## Discussion

By compiling the breadth and specific content of neonatal HIE registries across the world, we demonstrate considerable variability in the number, type, and format of the data collected by each registry. Variability of registry data collection likely reflects historical, geographical, and resource-driven factors, driven by local needs, priorities and capacities. Even when there were similarities among the types of data collected, the format in which it was collected often differed. Although not surprising given the lack of consensus in the field, our survey of the registries also confirmed variability in the population definitions used for registry inclusion and exclusion. Lastly, although many registries were developed at least in part to drive local quality improvement initiatives, nearly all reported that they also intended to use their data to support research efforts. To this end, improved harmonization of data elements and included neonates would allow for pooling of data between registries, supporting large-scale research.

The lack of consensus in definitions, inclusion criteria, and collected data throughout registries likely reflects differences in management across sites that have been previously documented in the literature by national and international surveys.^15,21^ These differences in management have also been linked to variations in outcomes in the context of HIE.^14^ Some of these differences may be related to potential differences in locally available resources. For example, the absence of neuroimaging data in 5 registries was unexpected, given the critical role neuroimaging plays in evaluating neonatal HIE and in understanding brain injury and recovery. Two of the registries lacking neuroimaging data were from middle-income countries, where neuroimaging resources may be more limited and therefore not identified as a primary focus in the data collection.

Identifying a set of core data elements can provide a foundation for data harmonization between studies, while still allowing for the collection of additional or specialized data elements relevant to the study’s specific goals. This approach would ensure both standardization and innovation, enabling registries to contribute to the broader research ecosystem, while still pursuing specialized inquiries. Collection of harmonized data from many diverse sites may leverage site variations in practices to design comparative effectiveness trials and to identify best care practices for these neonates.^5^ Identifying universally important data elements based on expert consensus and the literature and emphasizing elements critical for clinical care, benchmarking, and research will be the next important step. Such a systematic approach will be required to improve prognostic tools, tailor treatment strategies to disease phenotypes, and optimize outcomes and resource allocation.^22^

Registry data harmonization faces several challenges, including the following: (i) the need for consistent patient population definitions; (ii) divergent goals of registries resulting in variable interest in different types of data; (iii) lack of consensus on definitions of each data element; and (iv) individual registry concerns for proprietary collection tools. Although only one registry expressed discomfort releasing their data collection tool, we suspect that the number would be higher if we had requested inclusion of data into a common, central registry. Nevertheless, harmonization is vital for comparison of local, regional, national and international neonatal characteristics, practice and outcomes, especially in relatively rare diseases such as neonatal HIE. Group participation in analyzing and publishing results with authorship opportunities could serve as an incentive.

In recent years, multiple groups have advocated for improved robust and responsible data sharing following the findable, accessible, interoperable, and reusable principles^23^ through efforts such as the development of common data elements.^24,25^ As an example within the neurosciences, the National Institutes of Health National Institute of Neurological Diseases and Stroke has developed several sets of common data elements (CDEs) for neurological disorders such as cerebral palsy^26^ and stroke, in both adult and pediatric populations.^27^ Although work has begun to define core outcome measures for neonatal HIE,^28^ no CDEs are yet available for this population. The data from our study will ideally supplement and augment those from several individual groups who have already published and shared their data element sets^6,7,9,11,29^ as the first step in developing such CDEs; an effort which is currently ongoing within the Newborn Brain Society.

Even with published common data elements, however, the burden lies with the individual registries to implement those data elements, so the gold standard for data harmonization would be the development of a single central worldwide registry. Outside of NE/HIE, one such successful example of a large centralized registry with a narrow focus is the Extracorporeal Life Support Organization registry, which has collected data on over 49 000 neonatal extracorporeal membrane oxygenation runs since its inception,^30^ providing large quantities of extracorporeal membrane oxygenation-specific data to help optimize management and outcomes. Within NE/HIE, standardized data collection has been performed successfully in several moderately sized regional or national consortiums or networks, such as the Children’s Hospital Neonatal Consortium, Vermont Oxford Network, Canadian Neonatal Network, and the TOBY network. Although most of these network registries were not developed to study a specific disease but rather to collect relatively general data points on many neonatal diseases, valuable data can still be obtained from these types of large general registries.^7–9,14,15,31–34^ More detailed datasets and analyses will be necessary to advance the care of neonatal HIE in a significant way. Unfortunately, most attempts to develop HIE-specific registries (TOBY) or registry subsets (VON) have not been sustainable, in part due to difficulty securing long-term funding sources.

We acknowledge limitations to our study. Although we made every attempt to contact any registry that we could find through our literature search and discussions with international experts in the field, we may have not contacted every registry, and some registries chose not to or were unable to participate. Despite this limitation, the inclusion of registries from 14 countries representing 5 continents provides fairly wide population representation. Only 3 of the included registries were from LMICs and all 3 represented upper middle-income countries, so our results may be less generalizable to low- and lower middle-income countries. Additionally, some of the included registries were established prior to, or early in the establishment of TH as standard of care in HICs, so some variables may not have been updated to reflect the most up-to-date care. Lastly, some of the registries did not respond or responded incompletely to the survey regarding goals and definitions.

In conclusion, there is considerable variability in the number, type, and format of data collected in HIE registries maintained worldwide. Future attempts to develop standard common data elements to harmonize data collection could facilitate collaboration between registries and drive improvements in care for this high-risk population of neonates.

## Supplementary Material

Supplemental File 2

Supplemental File 1

Supplemental Table 1

Supp 5

Supplemental Table 2

## Figures and Tables

**Table I.** Number of registries from each country represented in the current survey, along with World Bank income classification

Income classification	Country	# Of registries*

High	United States of America	8
	Canada	3
	Australia	1
	Germany	1
	Israel	1
	Japan	1
	Netherlands	1
	New Zealand	1
	Spain	1
	Switzerland	1
	United Kingdom	1
Upper-middle	Brazil	1
	Malaysia	1
	Turkey	1

* Australia and New Zealand have a combined registry but are counted separately for this table.

**Table II.** Data element categories, with the median number of elements per category in each registry and the number of registries collecting any elements in that category, arranged from highest number of median elements per section to lowest

Category	Median # of related data elements (min, max)	Registries collecting data in category N (%)

Sociodemographics	2.5 (0,10)	21 (95.5)
Pregnancy	18 (1,49)	22 (100)
Labor, delivery, and sentinel events	4 (2,16)	22 (100)
Neonatal resuscitation	9.5 (3,22)	22 (100)
Acid/base status	6 (0,20)	20 (90.9)
Hypothermia data	9.5 (2,22)	22 (100)
Neonatal transport	4.5 (0,23)	21 (95.5)
Neurological examination	3 (0,24)	21 (95.5)
Neuroimaging	7 (0,21)	17 (77.3)
Neuromonitoring and seizures	7 (0,63)	20 (90.9)
Nonacid/base laboratory values	0 (0,60)	10 (45.5)
Hospital course management/outcomes	32 (6191)	22 (100)
Discharge status	8 (0,46)	21 (95.5)
Respiratory	6.5 (0,56)	19 (86.4)
Cardiovascular	4.5 (0,25)	21 (95.5)
Infectious	3.5 (0,30)	19 (86.4)
Feeding and nutrition	2 (0,21)	15 (68.2)
Hematologic	0.5 (0,15)	11 (50.0)
Renal and fluids	0.5 (0,8)	11 (50.0)
Gastrointestinal and hepatic	0 (0,7)	8 (36.4)

**Table III.** The top 22 individual data elements (defined as data elements reported by 13 or more registries), organized by the number (%) of registries collecting each data element (highest to lowest)

Data element	Registries collecting data element N (%)

Birth weight	22 (100)
Gestational age	22 (100)
1-min Apgar score	22 (100)
5-min Apgar score	22 (100)
Sex	20 (90.9)
Mode of delivery	20 (90.9)
10-minute Apgar score	19 (86.4)
Discharge disposition	17 (77.3)
Chest compressions in delivery room	16 (72.7)
Feeding status at discharge	16 (72.7)
Multiple birth	15 (68.2)
Chorioamnionitis/maternal infection	15 (68.2)
Age therapeutic hypothermia began	14 (63.6)
Maternal age	14 (63.6)
PPV received in delivery room	14 (63.6)
Intubation received in delivery room	14 (63.6)
Epinephrine received in delivery room	14 (63.6)
Length of hospital stay	14 (63.6)
Inborn	13 (59.1)
Prolonged rupture of membranes	13 (59.1)
Placental abruption	13 (59.1)
Persistent pulmonary hypertension	13 (59.1)
Magnetic resonance imaging performed	13 (59.1)

*PPV*, positive pressure ventilation.

## Data Availability

Data sharing statement available at www.jpeds.com.

## Newborn Brain Society Guidelines and Publications Committee:

Dr Sonia Bonifacio. Department of Pediatrics (Neonatology), Lucile Packard Children’s Hospital and Stanford University. Palo Alto, CA. United States; Dr Pia Wintermark. Department of Pediatrics, Faculty of Medicine and Health Sciences, McGill University. Montreal, Canada; Dr Hany Aly. Division of Neonatology, Cleveland Clinic Children’s Hospital, Cleveland, OH, United States; Dr Vann Chau. Department of Pediatrics (Neurology), The Hospital for Sick Children, SickKids Research Institute (Neuroscience and Mental Health) and University of Toronto, Ontario, Canada; Dr Hannah Glass. Departments of Neurology & Pediatrics, UCSF School of Medicine, University of California, San Francisco, San Francisco, CA, United States; Dr Monica Lemmon. Department of Pediatrics, Duke University School of Medicine, Durham, NC, USA, Department of Population Health Sciences, Duke University School of Medicine, Durham, NC, United States; Dr Courtney J. Wusthoff. Department of Neurology, University of California, Davis, CA, United States; Dr Gabrielle deVeber. Department of Pediatrics (Neurology), The Hospital for Sick Children, SickKids Research Institute (Child Health Evaluative Sciences) and University of Toronto, Toronto, Ontario, Canada; Dr Andrea C. Pardo. Department of Pediatrics (Neurology and Epilepsy). Northwestern University Feinberg School of Medicine. Chicago, IL, United States; Dr James P. Boardman. Centre for Reproductive Health, Institute for Regeneration and Repair, University of Edinburgh, Edinburgh, UK; Centre for Clinical Brain Sciences, University of Edinburgh, Edinburgh, United Kingdom; Dr Dawn Gano. Departments of Neurology & Pediatrics, UCSF School of Medicine, University of California, San Francisco, San Francisco, CA, United States; Dr Eric Peeples. Department of Pediatrics, University of Nebraska Medical Center, Children’s Nebraska, Child Health Research Institute, Omaha, NE, United States; Dr Lara Leijser. Department of Pediatrics, Section of Neonatology Cumming School of Medicine, University of Calgary, Alberta, Canada; Dr Firdose Nakwa. Department of Paediatrics and Child Health, Faculty of Health Sciences, University of the Witwatersrand, Johannesburg, South Africa; Dr Thiviya Selvanathan. Department of Pediatrics, BC Children’s Hospital Research Institute and University of British Columbia, Vancouver, BC, Canada.

## Abbreviations

CDE: Common data element
ECMO: Extracorporeal membrane oxygenation
HIC: High-income country
HIE: Hypoxic-ischemic encephalopathy
LMIC: Low- and middle-income countries
NE: Neonatal encephalopathy
PA: Perinatal asphyxia
TH: Therapeutic hypothermia
