# Supplementary material for: Data Collection Variability Across Neonatal Hypoxic-Ischemic Encephalopathy Registries
Source: J Pediatr. Author manuscript; Available in PMC 2026 Jul 21. (PMC13384812; doi:10.1016/j.jpeds.2025.114476)
Supplement: Supplemental File 2 [file NIHMS2186118-supplement-Supplemental_File_2.docx]

**List of Participating Registries (+ Key Personnel and/or Organization, if Different from Title)**

1. Australian & New Zealand Neonatal Network (Kei Lui, Rod Hunt)
2. Baby Cooling Registry of Japan (Japan Society of Perinatal and Neonatal Medicine and the Clinical Guidelines Committee for Neonatal Resuscitation in Japan)
3. Barcelona and Castilla y Leon Regional Spanish Registries (Alfredo García-Alix, Juan Arnaez)
4. California Perinatal Quality Care Collaborative (CPQCC)
5. Canadian Neonatal Network Database (Marc Beltempo)
6. Children’s Hospital Neonatal Database (Children’s Hospital Neonatal Consortium)
7. CRICO Neonatal Encephalopathy Registry (Mohamed El-Dib, Janet Soul)
8. Deutsche Hypothermieregister (Hemmen Sabir, Andreas Müller, Till Dresbach)
9. Florida Neonatal Neurologic Network, FN3 (Michael Weiss, Alfonso Varga, Jayne Solomon, Danilo Escoto, Jaime Flores-Torres)
10. HIE Surveillance Program (Khorshid Mohammad, Matthew Hicks, Stephen Wood, Amy Metcalfe)
11. Malaysian National Neonatal Registry (Perinatal Society of Malaysia)
12. Midwest Neonatal Encephalopathy Registry (Eric Peeples, Ann Anderson Berry, Howard Needelman)
13. NeoBrainLab_Asphyxia (Pia Wintermark)
14. Northeast Regional Therapeutic Hypothermia Database (Alexa Craig, Jay Kerecman, Deirdre O’Reilly, Matt Ryzewski)
15. National Cooling and Asphyxia Registry (Cornelia Hagmann, Barbara Brotschi)
16. Perined National Neonatal Registry/Landelijke Neonatale Registratie (Floris Groenendaal)
17. Protecting Brains & Saving Futures (Gabriel Fernando Todeschi Variane, Rafaela Fabri Rodrigues)
18. S-Neodata Database (Eilon Shany)
19. Seattle Children’s Hospital High-Risk Neonatal Database (Ulrike Mietzsch, Kendell German)
20. Turkish Neonatal Society Hypoxic Ischemic Encephalopathy Registry (Emel Okulu, Ibrahim Murat Hirfanoglu, Mehmet Satar, Omer Erdeve, Esin Koc)
21. The Vermont Oxford Network Neonatal Encephalopathy Registry (Vermont Oxford Network)
22. TOBY Cooling Register (Oxford Population Health National Perinatal Epidemiology Unit)
