# Supplementary material for: Data Collection Variability Across Neonatal Hypoxic-Ischemic Encephalopathy Registries
Source: J Pediatr. Author manuscript; Available in PMC 2026 Jul 21. (PMC13384812; doi:10.1016/j.jpeds.2025.114476)
Supplement: Supplemental File 1 [file NIHMS2186118-supplement-Supplemental_File_1.pdf]

Characteristics of Existing Registries for NE/HIE

Thank you and your team again for sharing the data entry form for your neonatal encephalopathy database. So far, we have compiled a total of 22 data forms from databases from around the world. The reason for this survey is to collect a few additional details from each of the registries who provided us with data forms.

Please complete the survey below.

Thank you!

Name of person completing this form

____

Name of data registry

____

What term is used in your registry to define the population?

- ◯ Neonatal encephalopathy
- ◯ Hypoxic-ischemic encephalopathy
- ◯ Asphyxia
- ◯ Other

Please describe which term is used

____

What definition is used to define whichever term you selected in the question above?

____

What are the inclusion criteria for your registry?

- ☐ All those treated with therapeutic hypothermia
- ☐ All those referred for potential therapeutic hypothermia
- ☐ Other

Provide detail of inclusion criteria used

____

Are there specific exclusion criteria for your registry?

- ◯ Yes
- ◯ No

What are the exclusion criteria?

____

What is/are the primary goal(s) of your registry?

- ☐ Local quality improvement initiatives
- ☐ Prospective or retrospective research
- ☐ Other

Describe other goals

____

Does your registry collect associated post-discharge follow up data?

- ◯ No
- ◯ Yes, as part of the same registry as the in-hospital data
- ◯ Yes, as a separate but connected registry dedicated to follow up data

Up to what age (years) do you collect follow up data?

____

Would you be willing to share the data elements for your separate follow up registry?

◯ Yes
◯ No

What is the geographical coverage of your registry (i.e. how many cities are covered by the included sites)?

____

How many hospitals enter data in your registry?

____

How many of those sites provide each of the following care to neonates:

____

Therapeutic hypothermia to neonates with HIE?

Non-invasive CPAP?

____

Intubation and mechanical ventilation?

____

High-frequency ventilation (HFOV/HFJV)?

____

ECMO?

____

Surgery?

____

What is/are the funding source(s) for your registry?

☐ None
☐ Internal grant
☐ Foundation grant
☐ Philanthropic grant
☐ Insurance company
☐ National grant (NIH, ERC, PERF, etc)
☐ Other

Please describe your other funding source

____

Do any of your sites use electronic health records?

◯ Yes
◯ No

Which one(s)?

____

Would you be interested in potentially harmonizing your database to integrate common data elements that will be agreed upon by the participating databases?

◯ Yes
◯ No
