# Supplementary material for: Data Collection Variability Across Neonatal Hypoxic-Ischemic Encephalopathy Registries
Source: J Pediatr. Author manuscript; Available in PMC 2026 Jul 21. (PMC13384812; doi:10.1016/j.jpeds.2025.114476)
Supplement: Supplemental Table 1 [file NIHMS2186118-supplement-Supplemental_Table_1.pdf]

Variable	Definition	Variable Type (Num, MC, Free, D/T)
**Demographics**		
Maternal age		Num
Advanced maternal age		MC
Maternal Race		MC
Ethnicity		MC
Australian indigenous status of mother		MC
Mother highest grade		MC
Highest level of education parent 1		Free
Highest level of education parent 2		Free
Primary caregiver's preferred language		MC
Did the primary caregiver require interpreter services		MC
Mother's marital status		MC
Neonate Sex		MC
Neonate Race		MC
Ethnicity		MC
Jewish		MC
Insurance		MC
**Pregnancy**		
Source of referral		MC
Prenatal care		MC
Antenatal care <20 weeks		MC
Early US		MC
GA when US done		Num
Date of conception (if IVF)		D/T
LMP		D/T
Menstrual cycle length	Days	Num
Details of antenatal care		Free
Current pregnancy details		Free
Assisted reproduction	Pregnancy was the product of artificial or assisted reproductive technology. This may include medications to induce ovulation (clomid) and/or procedures to assist fertilization (artificial insemination, in vitro fertilization).	MC
Gravida		Num
How many full term pregnancies (including this one)		Num
How many preterm pregnancies (including this one)		Num
Para		Num
Previous abortion		Num
Previous perinatal death		MC
Previous preterm birth		MC
Living children		Num
Multiple birth		MC
Birth order of this infant		Num
Placentation		MC
Mode of delivery		MC
Presentation		MC
Previous C-section		MC
Indication for admission for delivery		MC
Vaginal bleeding		MC
Delivery induced		MC
Duration of dilation phase	Minutes	Num
Duration of delivery		Num
Anesthesia		MC
Risk for neonatal infection		MC
PPROM		MC
Rupture of membranes duration		Num
Rupture of membranes ≥18 or 24 hours		MC
Sentinel events		MC
Sentinel event		Free
Cord prolapse		MC
Cord avulsion		MC
Tight nuchal cord		MC
True knot in cord		MC
Maternal cardiac arrest/CPR		MC
Uterine rupture		MC
Head entrapment		MC

Difficult extraction		MC
Vacuum extraction		MC
Shoulder dystocia		MC
Duration	Minutes	Num
Placental abruption		MC
Placenta previa		MC
Vasa previa		MC
Placenta accreta/increta/percreta		MC
Feto-maternal hemorrhage		MC
Maternal event		MC
Maternal trauma		MC
Maternal hypotension		MC
Maternal hypoglycemia		MC
Amniotic embolism		MC
Antepartum hemorrhage	Placenta previa, abruption or threatened abortion resulting in bleeding that can be external (vaginal bleeding in the absence of a vaginal or cervical source) or occult (retroplacental clot) other than bloody show was documented after 20 weeks of pregnancy in the maternal or infant medical record.	MC
Chorioamnionitis/infection		MC
Describe antenatal infection		Free
Risks for clinical chorioamnionitis		MC
Foul smelling fluid?		MC
Maternal fever (>38.0C) during labor		MC
Maternal fever (≥38.5C) during labor prior to delivery		MC
Maternal fever (≥38.5C) after delivery		MC
Duration of maternal fever		Num
Maximum temperature	The highest maternal temperature recorded during labor in degrees Centigrade, as documented during labor in the maternal or infant medical record	Num
Antipyretics given		MC
Maternal WBC count		Num
Antepartum or intrapartum antibiotics		MC
If yes, which?		MC
How many doses		Num
Administered how long before delivery		Num
Antenatal steroids (betamethasone)		MC
Most recent course		MC
How many doses		MC
More than one course?		MC
Antenatal magnesium exposure		MC
Other intrapartum medications		MC
Any pregnancy complications		MC
Maternal hypertension	Maternal hypertension, chronic or pregnancy induced, with or without edema and proteinuria, was recorded in the maternal or infant medical record, or if a maternal blood pressure above 140 systolic or 90 diastolic was not recorded prior to or during the present pregnancy	MC
Pre-eclampsia	Montreal: Blood pressure >= 140/90 mmHg or increased systolic blood pressure30mmHg or increased diastolic blood pressure 15mmHg starting at or after 20 weeks ofgestation	MC
Eclampsia		MC
HELLP		MC
Hypertension chronicity		MC
Maternal diabetes	Maternal diabetes mellitus treated with insulin and/or an oral hypoglycemic agent during or prior to the present pregnancy was recorded in the maternal or infant medical record; Montreal defined No = NPO blood sugar <6 mmol/L or 2h after meal blood sugar <7.8mmol/L; Intolerance= NPO blood sugar between 6-7 mmol/L or 2h after mealblood sugar between 7.8-11.1 mmol/L; Diabetes= NPO blood sugar > 7 mmol/L or 2h after meal blood sugar>11.1 mmol/L or diabetes diagnosed before pregnancy	MC
Diabetes chronicity		MC
Maternal epilepsy		MC

Maternal cardiac problems		MC
Maternal thyroid disease		MC
Maternal severe anemia		MC
Pre-pregnancy obesity (BMI>30)		MC
Fetal growth restriction/IUGR		MC
Polyhydramnios	Volume of amniotic fluid > 2L or Index amniotic fluid >18-20cm	MC
Oligohydramnios	Volume of amniotic fluid < 250mL or amniotic fluid index < 5cm	MC
Maternal depression and/or anxiety		MC
Maternal medications (other than PNV)		MC
Number of maternal medications		Num
List maternal medications		Free
Maternal smoking/tobacco		MC
Packs per day		Num
Maternal alcohol use		MC
Number of drinks/week		Num
Maternal substance use		MC
Which illicit drugs were used?		MC
Specify other illicit drugs used		Free
How long since illicit drugs taken	days	Num
Urine toxicology screen obtained?		MC
Toxicology positive for which drugs		MC
Specify other positivity on toxicology		Free
Maternal blood type (ABO)		MC
Maternal blood type (Rh)		MC
GBS positive		MC
RPR/VDRL positive		MC
Hep B positive		MC
HIV positive		MC
Rubella		MC
Prenatal TORCH infection present		MC
Toxoplasmosis		MC
Rubella		MC
Syphilis		MC
CMV		MC
Herpes Simplex		MC
Prenatal genetic testing		MC
Other maternal illness		Free
**Delivery**		
Any delivery complications		MC
Intrauterine pH		Num
Fetal heart rate monitoring		MC
Non-reassuring fetal status		MC
Fetal monitoring results		MC
Abnormal FHR		MC
Bradycardia	Baseline fetal heart rate < 110 beats per minute on fetal heart rate monitoring (electronic or auscultation) prior to delivery	MC
Tachycardia	Baseline fetal heart rate >160 beats per minute on fetal heart rate monitoring prior to delivery (electronic or auscultation) was noted	MC
Decreased FHR variability	Minimal (amplitude range detectable but less than 5 beats per minute or fewer) or absent (amplitude range undetectable) fetal heart rate variability on electronic fetal heart rate monitoring prior to delivery was noted	MC
Prolonged or recurrent decelerations		MC
Early decelerations		MC
Variable decelerations		MC
Late decelerations		MC
Category 2 trace		MC
Category 3 trace		MC
Upload fetal rhythm strip prior to delivery		Free
Amniotic fluid		MC
Meconium-stained amniotic fluid		MC
Amount		MC
Placenta sent for pathology		MC
Copy results		Free

Placenta weight		Num
Fetal thrombotic vasculopathy		MC
Villitis of unknown etiology		MC
Chorioamnionitis		MC
Chorioamnionitis with fetal vasculitis		MC
Chorioamnionitis without fetal vasculitis		MC
Chorionic plate meconium		MC
Traumatic birth injury		MC
Birth injury		MC
Subdural and/or epidural bleeding		MC
Subgaleal bleeding		MC
Laceration head		MC
Spinal cord lesion		MC
Spinal cord injury		MC
Brachial plexus injury		MC
Fracture		MC
Skull fracture		MC
Long bone fracture		MC
Clavicle fracture		MC
Cephalohematorma		MC
Other		MC
Birth weight	Grams	Num
Percentile		MC
Birth length	cm	Num
Percentile		MC
Head circumference	cm	Num
Percentile		MC
Gestational age	weeks, days	Num
Based on		MC
Neonatal estimate of GA	weeks, days	Num
Prematurity		MC
Postmaturity		MC

**Neonatal Resuscitation/Delivery Room**		
Resuscitation required		MC
Delayed cord clamping		MC
Time to cord clamping	Seconds	Num
Cord milking		MC
Details of delivery and resuscitation		Free
Oxygen required		MC
Highest fiO2 during resuscitation		Num
FiO2 at start of resuscitation		Num
Saturations at start of resuscitation		Num
FiO2 at end of resuscitation		Num
Saturations at end of resuscitation		Num
CPAP required		MC
PPV required		MC
Duration of PPV	Minutes	Num
≥10 min of life		MC
Nasal ventilation required		MC
Airway management		MC
Time to advanced airway placement		MC
LMA used		MC
Intubation required		MC
Extubation in delivery room		MC
Age of extubation	Minutes	Num
Ventilation required at end of resuscitation		MC
Assisted ventilation at 10 min		MC
Type of respiratory support at 10 min		MC
Continue resuscitation after 10 min		MC
Chest compressions required		MC
Duration of chest compressions	Minutes	Num
Provider performing compressions		MC
History of asystole in the delivery room		MC
Time of heart rate recovery	Minutes	Num
UVC placed in delivery room		MC
Resuscitation medications required in the delivery room		MC
Select medications given		MC
Epinephrine required in delivery room		MC
Time to first dose		Num

Total epi dose given	ml/kg	Num
Total number of epi doses given		Num
Route of epinephrine		MC
NaCl bolus in delivery room		MC
Volume of fluid expansion given	ml/kg	Num
Volume of sodium bicarb given in delivery room	mEq/kg	Num
pRBC transfusion in delivery room		MC
Volume of pRBC transfusion in delivery room		Num
Apgar score at 1 min		Num
Apgar score at 5 min		Num
Apgar score at 10 min		Num
≤5?		MC
Apgar score at 15 min		Num
Apgar score at 20 min		Num
Asphyxia?	Must meet three criteria: 1)late decelerations meconium stained amniotic fluid; 2) need for respiratory support, 3) umbilial arterial cord pH < 7.1, 4) 5-min Apgar < 7, 5)multi-organ failure	MC
Spontaneous activity at birth		MC
First spontaneous breath after birth	Minutes	Num
Total duration of resuscitation	Minutes	Num
Died in the delivery room		MC
**Acid/base Status**		
Cord blood gas obtained?		MC
Source of blood gas		MC
pH from cord blood sample	If either arterial or venous umbilical cord blood sampling was performed, record the pH observed to two decimal places. If multiple umbilical cord samples were obtained, record the lowest pH observed to two decimal places.	Num
Base deficit from cord blood sample	If either arterial or venous umbilical cord blood sampling was performed, record the base deficit observed in mmoles/liter to the nearest tenth. If multiple umbilical cord samples were obtained, record the worst (highest) base deficit measured or calculated in mmoles/liter.	Num
pCO2 from cord blood sample		Num
pO2 from cord blood sample		Num
HCO3 from cord blood samples		Num
Lactate from cord blood sample		Num
UA pH		Num
UA paCO2		Num
UA paO2		Num
UA HCO3		Num
UA BD		Num
UA lactate		Num
UV pH		Num
UV paCO2		Num
UV paO2		Num
UV HCO3		Num
UV BD		Num
UV lactate		Num
Arterial cord pH <7.0 and/or cord BE ≤ -16		MC
Postnatal gas (<1 hr of age) obtained	Montreal collected first postnatal regardless of time	MC
Type of blood gas		MC
pH from blood gas in first hour of life	Record the pH obtained in the first hour of life to two decimal places. If multiple samples were obtained, record the lowest pH observed to two decimal places.	Num
Base deficit from blood gas in first hour of life	Record the base deficit obtained in the first hour of life to two decimal places. If multiple samples were obtained, record the worst (highest) base deficit observed to two decimal places.	Num
pH<7.0 and/or cord BE ≤ -16		MC
Cord or first arterial pH (worse of the two)		Num
Cord or first arterial BD (worse of the two)		Num
Cord or first arterial paO2 (worse of the two)		Num
Cord or first arterial paCO2 (worse of the two)		Num
Cord or first arterial lactate (worse of the two)		Num

Postnatal pH		Num
Postnatal BD		Num
Postnatal HCO3		Num
Postnatal lactate		Num
Postnatal pCO2		Num
Postnatal pO2		Num
Lowest pH	D1, 2, 3, 4	Num
Lowest paO2	D1, 2, 3, 4	Num
Highest paO2	D1, 2, 3, 4	Num
Lowest pCO2	D1, 2, 3, 4; 0-6hrs, 0-72 hrs	Num
Highest pCO2	D1, 2, 3, 4; 0-6 hours, 0-72 hours	Num
Anion gap		Num
Peak lactate	Calgary restricted to first 24 hours	Num
Date of test		D/T
**Transport**		
Teleconsult obtained		MC
Transport data available		MC
Inborn		MC
If outborn location of birth		MC
If outborn, method of transport		MC
If outborn, did delivery hospital have level 3 NICU		MC
If outborn, distance of transport	km	Num
If outborn, unit where baby located in transferring hospital		MC
Reason for transfer		MC
Transport team		MC
Transport provider		MC
Hours between decision to transfer and arrival at referral center		Num
Transferring hospital	Enter the name and location of the hospital from which the infant was transferred. For location, U.S. hospitals record city and state; Canadian hospitals record city and province; international centers record city and country.	Free
Birth hospital annual birth rate		MC
Pediatrician in house		MC
Transport heart rate (prior to transport)	BPM	Num
Transport systolic BP (prior to transport)	mm Hg	Num
Transport diastolic BP (prior to transport)	mm Hg	Num
Hypothermia at transferring hospital		MC
Date and time cooling started		D/T
Type of cooling at transferring hospital		MC
Method(s) used for maintaining hypothermia		MC
Was the baby overcooled at referral site		MC
Time to stabilization	Minutes from birth until transport team loads patient for departure	Num
Time from transport team arrival to reaching goal temp		Num
Hypothermia during transport		MC
Method of transport		MC
Type of cooling during transport		MC
Method(s) used for maintaining hypothermia		MC
Date and time transport cooling started		D/T
Highest temperature during transport		Num
Lowest temperature during transport		Num
Responsiveness at transport team arrival		MC
Heart rate at transport team arrival		Num
Respiratory rate at transport team arrival		Num
Oxygen saturation at transport team arrival		Num
Respiratory status at transport team arrival		MC
Inspired oxygen concentration at transport team arrival		Num
Respiratory support at transport team arrival		MC
Blood pressure at transport team arrival		Num
Temperature measurement method by transport team		MC
Temperature measurement frequency in transport		MC
Temperature at transport team departure from transferring hospital		Num
Temperature during transfer (1)		Num
Temperature during transfer (2)		Num
Temperature at transport team arrival to receiving hospital		Num
Responsiveness at transport team arrival to receiving hospital		MC
Heart rate at transport team arrival to receiving hospital		Num
Respiratory rate at transport team arrival to receiving hospital		Num

Oxygen saturation at transport team arrival to receiving hospital		Num
Respiratory status at transport team arrival to receiving hospital		MC
Inspired oxygen concentration at transport team arrival to receiving hospital		Num
Respiratory support at transport team arrival to receiving hospital		MC
Blood pressure at transport team arrival to receiving hospital		Num
Was target temp maintained during transport		MC
Was the baby overcooled on transport		MC
Significant events during transfer		Free
Type of drugs and dosage during transfer		Free
Age at admission to referral center		Num
Weight at admission to referral center	grams	Num
**Hypothermia Data**		
Less than 6 hours old		MC
Met criteria **to consider** TH	1- > or =34 GA and up to 12 hrs AND 2- Any one of: sentinel event prior to delivery(Uterine rupture, profound bradycardia or cordprolapse) or Apgar @10min< or =5, or prolongedresuscitation at 10 min of life (Chest compression,intubation, and or mask ventilation at 10 min) , orpH< 7.1, within 60 min of life, or BD < or = 10 within60 min of life, post-natal collapse resulting inhypoxia -Ischemic injury.	MC
Met criteria for TH	1- > or =36 GA and < 6 hrs AND 2- Any one of: sentinel event prior to delivery(Uterine rupture, profound bradycardia or cordprolapse) , or Apgar @10min< or = 5, or prolongedresuscitation till 10 min of life (Chest compression,intubation, and or mask ventilation at 10 min), orpH< 7.0, within 60 min of life, or BD < or = 16 within60 min of life.	MC
Hypothermia qualification		MC
Exclusion criteria		MC
Was the baby treated with therapeutic hypothermia?		MC
Other neuroprotective strategies?		Free
If transported, was hypothermia continued at receiving hospital		MC
Cooling method at receiving hospital		MC
Cooling equipment used		MC
Temperature measurement method		MC
Temperature at start of resuscitation		Num
Temperature during resuscitation		Num
Temperature at end of resuscitation		Num
Warmer heat turned off during resuscitation?		MC
Temperature measured within 1 hour of arrival		MC
Temperature on admission	Degree C with one decimal	Num
Temp too low		MC
First temperature prior to therapeutic hypothermia		Num
Highest infant temperature in first 72 hours		Num
Lowest infant temperature in first 72 hours		Num
Age hypothermic therapy began	Hours, minutes	Num
Date and time first reached goal temp (33-34C)		D/T
Target temperature		Num
Lowest recorded temperature during hypothermia		Num
Highest recorded temperature during hypothermia		Num
Was temperature <33C		MC
Was temperature >34C		MC
Temperature during hypothermia		Num
Respiratory support required during cooling		MC
Received sedation before start of cooling		MC
Type of drugs and dosage		Free
Received sedation during active TH		MC
Method of sedation received during TH		MC
Sedatives given before grading HIE		MC
Infusion detail		MC
Highest infusion rate of sedation		Num
Day of life sedation was discontinued		Num
Bolus details		MC
Date and time cooling stop		D/T

Core body temperature when hypothermic therapy stopped	Record the core body temperature measured closest in time to when hypothermic therapy stopped and the units in which it was recorded (Centigrade). Record rectal, esophageal, tympanic or axillary temperature using a single decimal place, e.g. 36.7° C. If cooling is interrupted and is re-started, record the temperature when hypothermic therapy was finally stopped.	Num
Duration of hypothermia	Hours	Num
Prolonged hypothermia administered		MC
Was there early exit from TH		MC
Reason for early exit		MC
Was cooling interrupted for >30 min		MC
Number of interruptions		Num
Age rewarming began	Days, hours, minutes	Num
Warming systolic BP		Num
Warming diastolic BP		Num
Warming HR range		Free
Warming resp range		Free
Mechanical ventilation during warming		MC
Ventilation rate		Num
Age rewarming completed	Days, hours, minutes	Num
Was the baby held during cooling		MC
Age at first holding by parents		Num
Adverse events of hypothermia within 7 days of birth		
Cardiac arrhythmia	persistent or recurrent arrhythmia excluding sinus bradycardia, or requiring treatment of re-warming (CRICO)	MC
Bradycardia		MC
Thrombosis	Not related to an infusion line	MC
Septicemia	blood-culture-positive septicaemia	MC
Intractable acidosis		MC
Electrolyte imbalance	Na, Ca, K, Phos	MC
Liver dysfunction		MC
Hypoglycemia	blood glucose <45mg/dL; <2.6 mmol/litre	MC
Severe hypotension	Requiring treatment with pressors or hydrocortisone or persistent hypotension with mean blood pressure ≤40mmHg	MC
Seizures during re-warming	Clinical or EET seizures	MC
Scalp edema		MC
Skin breakdown		MC
Sclerema neonatorum/subcutaneous fat necrosis		MC
Thrombocytopenia		MC
Syndrome of inappropriate secretion of ADH		MC
Cerebral salt wasting		MC
Severe bleeding/coagulopathy		MC
Necrotizing enterocolitis		MC
Concern for other side effects of TH		MC
Describe other side effect		Free

Neurological Examination

Moribund on admission		MC
Head circumference - 1 week of age	cm	Num
Head circumference - 2 week of age	cm	Num
Head circumference - 3 week of age	cm	Num
Date and time of encephalopathy exam (1st, 2nd, 3rd, etc)		D/T
Was encephalopathy exam repeated		MC
Number of encephalopathy scores taken		Num
Date and time of worst NE in the first 12 hours		D/T
Alive at this time point		MC
Paralysis for entire time period		MC
Neurological exam prior to hypothermia		MC
Who performed the exam		MC
Type of exam being documented		MC
Modified Sarnat stage prior to cooling	Based on the predominant number of categories	MC
Modified Sarnat stage at start of hypothermia		MC
Modified Sarnat stage at 3-6 hours of life		MC
Modified Sarnat stage at completion of hypothermia		MC
Modified Sarnat stage at referral, admission, day 1/2/3, day 4/5		MC
Thompson score on admission		Num
Thompson score at 24, 48, 72h		Num

Highest Thompson score		Num
Thompson Tone		MC
Thompson LOC		MC
Thompson fits		MC
Thompson posture		MC
Thompson Moro		MC
Thompson grasp		MC
Thompson suck		MC
Thompson resp		MC
Thompson fontanel		MC
Level of consciousness	Select the worst conscious state during the period (from best to worst, states are normal, irritability, lethargy, stupor and coma): Answer “Normal” if the infant is in the normal conscious state during the entire examination period. Answer “Irritability” if there is excessive, often high-pitched cry with little sustained quiet (which may be associated with spontaneous tremors). Answer “Lethargy” if the infant sleeps excessively with occasional spontaneous eye opening. Answer “Stupor” if there is no spontaneous eye opening, and tactile stimulation elicits poorly sustained eye opening. Answer “Coma” if there is no eye opening with vigorous tactile stimulation ***need additional descriptions for other states***	MC
Brainstem function	Answer“Normal” if brainstem function was normal during the entire examination period.Answer“Abnormal” if any of the following abnormalities were observed at any time during the examination period: decreased facial movements; eyes not normally aligned or incomplete eye movements to Doll’s eye maneuver; poor suck, excess drooling, decreased swallow, or depressed gag reflex; depressed corneal reflex; sustained periodic hyperventilation or ‘gasping’ patterns.	MC
Spontaneous activity	Answer“Normal” if the infant’s movements were normal in frequency and amplitude throughout the examination period.Answer“Abnormal” if the infant’s movements were decreased in frequency or amplitude at any time during the examination period. ***need additional descriptions for other states***	MC
Posturing	Posturing is sustained abnormal position such as tonic extension or flexion of limbs or trunk, e.g., opisthotonus (extension of neck and trunk); retrocollis (extension of neck); decerebrate posturing (extension of arms and legs); decorticate posturing (extension of legs/ flexion of elbows).	MC
Posture		MC
Tone	Tone is abnormal if it is either increased (shows increased resistance to passive movement) or decreased (shows decreased resistance to passive movement, which may be associated with frog-leg posturing with arms and legs lying flaccid in abduction). ***need additional descriptions for other states***	MC
Adjunct tone information		MC
Reflexes	Reflexes are abnormal if they (1) are hyperactive e.g., exaggerated response to tendon tap (which may be associated with sustained clonus of > 5 beats at the ankles) or (2) show crossed adductor response of opposite knee when tapping medial aspect of knee or (3) are hypoactive, e.g., when there is reduced or absent response to tendon tap.	MC
Sucking reflex		MC
Grasp reflex		MC
Moro reflex		MC
Gag reflex		MC
Pupil light reflex		MC
Pupil doll's eye		MC

Heart rate		MC
Respiration		MC
Mechanically ventilated during assessment		MC
Feeding prior to cooling		MC
Spanish scoring system - total		Num
Seizures prior to cooling		MC
Type of seizures		MC
HIE?	within seven days of birth	MC
HIE severity		MC
**Laboratory values**		
CBC date/time		Num
Platelet count		Num
Highest platelets recorded		Num
Lowest platelets recorded		Num
Coags date/time		D/T
PT		Num
PT activity (%)		Num
<60%		MC
≤40%		MC
≤20%		MC
Fibrinogen		Num
aPTT		Num
D-dimer		Num
INR		Num
Highest PT		Num
Highest INR		Num
Highest PTT		Num
Lowest fibrinogen		Num
Hematocrit		Num
Highest hematocrit		Num
Lowest hematocrit		Num
Hemoglobin		Num
Infant blood type		MC
Rh		MC
Cortisol		Num
Cardiac enzymes date/time		Num
Creatine kinase (CK)		Num
CK-MB		Num
Troponin		Num
BNP obtained?		MC
Value (up to first 10 values)		Num
LDH		Num
Initial nRBC count		Num
Date of test		D/T
Highest nRBC		Num
Electrolytes date/time		D/T
Potassium		Num
Chloride		Num
HCO3		Num
Magnesium		Num
Phosphorus		Num
Sodium		Num
Initial Na		Num
Lowest Na		Num
Highest NA		Num
Creatinine		Num
Initial creatinine		Num
Date of test		D/T
Peak creatinine		Num
Date of test		D/T
Blood urea nitrogen		Num
Cystatine C		Num
Ammonia		Num
Total protein		Num
Albumin		Num
B2-microglobin		Num
NSE		Num
S100B		Num
Highest amikacin level		Num
Lowest glucose		Num

Highest glucose	Num
Glucose on admission	Num
Glucose	Num
Calcium	Num
Lowest total calcium	Num
Highest total calcium	Num
Lowest ionized calcium	Num
Highest ionized calcium	Num
LFT date/time	D/T
Total bilirubin	Num
Conjugated bilirubin	Num
Alkaline phosphatase	Num
AST	Num
<100	MC
100-500	MC
>500	MC
ALT	Num
<100	MC
100-500	MC
>500	MC
Initial AST	Num
Date of test	D/T
Peak AST	Num
Date of test	D/T
Initial ALT	Num
Date of test	D/T
Peak ALT	Num
Date of test	D/T
GGT	Num
WBC	Num
Highest WBC	Num
Lowest WBC	Num
Bands	Num
Highest I:T ratio	Num
Lymphocytes	Num
Procalcitonin	Num
CRP	Num
Units used	MC
Date of test	D/T
Urine blood	Free
Urine protein	Free
Urine specific gravity	Free
Urine osmolality	Free
Urine Na	Free
Urine K	Free
CSF - age obtained	Num
Successful lumbar puncture?	MC
Contraindicated (instability or bleeding disorder)?	MC
CSF - hemorrhagic appearance?	MC
CSF glucose	Num
CSF protein	Num
CSF WBC	Num
CSF RBC	Num
CSF RBC/WBC	Num
CSF albumin	Num
CSF B2-microglobulin	Num
CSF neopeterin	Num
CSF NSE	Num
Postnatal genetic testing obtained	MC
What was sent	Free
What was sent	MC
Metabolic testing sent	MC
If sent, results?	MC
If abnormal, paste results	Free
Was an underlying genetic cause for encephalopathy determined	MC
What was the final diagnosis	Free
Cord toxicology screen	MC
Urine toxicology screen	MC
Meconium toxicology screen	MC
Newborn Screen 1,2,3	MC

Neuromonitoring/Seizures		
Full channel EEG prior to cooling		MC
EEG background prior to cooling		MC
aEEG prior to cooling		MC
aEEG background prior to cooling		MC
Possible seizures on aEEG prior to cooling		MC
Full channel EEG performed		MC
Date/time cEEG commenced		D/T
Date/time cEEG ended		D/T
cEEG impression		MC
cEEG findings at beginning of recording		MC
cEEG findings 24 hours of recording		MC
cEEG findings 48 hours of recording		MC
cEEG findings 72 hours of recording		MC
cEEG findings at end of recording		MC
cEEG Summary report		MC
cEEG initial 24 hour report		Free
EEG background pattern	If a full channel EEG was performed at any time prior to discharge from your hospital, select the one category that most closely describes the worst EEG background pattern observed on any full channel EEG prior to discharge from your hospital, including EEGs performed prior to admission for outborn infants (from best to worst, patterns are normal, excessively discontinuous, depressed amplitude, burst suppression and background iso-electric).Answer“Normal” if the background pattern is normal in continuity, amplitude and frequency for gestational age.Answer“Excessively Discontinuous”if the background pattern is excessively discontinuous (‘dysmature’) for gestational age.Answer“Depressed Amplitude”if the background pattern shows depressed amplitude and/or slowing.Answer“Burst Suppression Pattern”if a burst suppression pattern is present or a severely depressed background is present.Answer“Background Iso-Electric”if the background pattern shows no recognizable electro-cortical activity.	MC
Worst grade of NE assigned by cEEG		MC
Sleep wake cycling on EEG		MC
Age that sleep wake cycling occurred		MC
Abnormal EEG at 7-14 days		MC
Abnormal EEG results		Free
Bedside aEEG performed		MC
Date/time aEEG commenced		D/T
Date/time aEEG ended		D/T
Bedside aEEG pattern	Answer“Normal” if the upper margin of the dense aEEG band is greater than 10µv and the lower margin is greater than 5µv.Answer“Moderately Abnormal or Discontinuous” if the upper margin of the dense aEEG band is greater than 10µv and the lower margin is less than 5µv. Answer“Severely Abnormal” if the upper margin of the dense aEEG band is less than 10µv and the lower margin is less than 5µv.	MC
aEEG interpreter		MC
Worst aEEG background activity		MC
Time to normal aEEG tracing	hours	Num
Appearance of sleep-wake cycling on aEEG	hours	Num
Well established sleep-wake cycling on aEEG	hours	Num
Monitoring indication		Free
aEEG pattern during rewarming		MC
aEEG interpreter		MC

Clinical seizures	A clinical seizure is defined as “paroxysmal tonic, clonic or myoclonic motor activity that cannot be suppressed by restraint or repositioning and/or if there are paroxysms of abnormal oromotor or oculomotor activity which may be associated with changes in autonomic function (otherwise unexplained paroxysmal tachycardia/hypertension/papillary dilation)”	MC
Character of clinical seizures		MC
Any electrographic seizure		MC
Full channel EEG evidence of seizures		MC
Bedside aEEG evidence of seizures		MC
Electroclinical correlation		MC
Any seizures requiring treatment?	clinical and/or electrographic	MC
Type of seizures	Answer“Yes” if one or more seizures were documented in the infant medical record at any time prior to discharge from your hospital. The diagnosis may be made by clinical observation, full channel EEG or bedside aEEG monitor.Answer“No” if one or more seizures were not documented in the infant medical record at any time prior to discharge from your hospital.	MC
Date/time of first seizure		D/T
Age seizures first observed	Days, hours, minutes	Num
Date/time of last seizure		D/T
Duration of seizures	minutes	Num
Description of seizures		MC
Status epilepticus		MC
Anticonvulsants prior to discharge		MC
Number of anticonvulsants		Num
Loading Dose only		MC
Maintenance therapy		MC
Phenobarbital		MC
First loading dose		MC
Second loading dose		MC
Start date/time		D/T
End date/time		D/T
Starting age		Num
Loading dose amount	mg/kg	Num
Maintenance dose	mg/kg	Num
Maintenance interval		Num
Completion age		Num
Total cumulative dose	mg/kg	Num
Level value		Num
Age when level obtained		Num
Phenytoin or fosphenytoin		MC
Start date/time		D/T
End date/time		D/T
Starting age		Num
Loading dose	mg/kg	Num
Maintenance dose	mg/kg	Num
Maintenance interval		Num
Completion age		Num
Total cumulative dose	mg/kg	Num
Level value		Num
Age when level obtained		Num
Lorazepam		MC
Diazepam		MC
Midazolam		MC
Start date/time		D/T
End date/time		D/T
Starting age		Num
Loading dose	mg/kg	Num
Completion age		Num
Total cumulative dose	mg/kg	Num
IV infusion	mg/kg/h	Num
Topiramate		MC
Oxcabamazepine		MC
Lacosamide		MC
Levetiracetam		MC
Start date/time		D/T

End date/time		D/T
Starting age		Num
Loading dose	mg/kg	Num
Maintenance dose	mg/kg	Num
Maintenance interval		Num
Completion age		Num
Total cumulative dose	mg/kg	Num
Level value		Num
Age when level obtained		Num
Pyridoxine		MC
Start date/time		D/T
End date/time		D/T
Lidocaine		MC
Starting age		Num
Loading dose	mg/kg	Num
Completion age		Num
Total cumulative dose	mg/kg	Num
IV infusion	mg/kg/h	Num
Level value		Num
Age when level obtained		Num
Other anticonvulsants		MC
Duration of treatment with anticonvulsants	days	Num
Number of antiseizure medications		MC
What type of seizures were anticonvulsants started for		MC
NIRS monitoring?		MC
Time of starting NIRS monitor		D/T
Age in hours of NIRS monitor start		Num
Time of ending NIRS monitor		D/T
Total NIRS recording duration	hours	Num
NIRS monitor sensor		Free
NIRS type/location		Free
NIRS high (≥85%)		MC
NIRS lacking variability (<5%)		MC
Cerebral NIRS range		MC
Hour measurement was taken		Num
Upload NIRS log		Free
**Neuroimaging**		
Day of first imaging		Num
Imaging type		MC
Results		Free
Day of last imaging exam		Num
Any abnormal imaging findings (HUS/MRI)?		MC
Any hemorrhage		MC
Intraventricular hemorrhage		MC
Grade of IVH	I, II, III, IV	Num
Extra-axial, subdural or subarachnoid hemorrhage		MC
Intraparenchymal hemorrhage		MC
Subependymal hemorrhage		MC
Abnormality in deep nuclear gray matter/BGT		MC
Watershed injury/white matter		MC
Cystic white matter injury		MC
Diffuse white matter injury		MC
Diffusion changes		MC
Spectroscopic changes		MC
Ventriculomegaly		MC
Focal ischemia/Stroke		MC
Veno-occlusion		MC
Arterial occlusion		MC
Arterial Infarct		MC
Multifocal embolic infarcts		MC
Cerebellar parenchymal injury		MC
Other cerebral parenchymal injury?		MC
Other intracranial abnormalities		MC
Head US performed		MC
Date of first Head US		D/T
Age of first head US		Num
Head US normal		MC
Result first head US		Free
Result of each head US		MC

Was a second head US performed		MC
Date of head US 2		D/T
Result head US 2		Free
Impaired white/grey matter differentiation and/or slit like ventricles		MC
Hyperechogenicity periventricular white matter		MC
Peri-intraventricular hemorrhage		MC
Hyperechogenicity subcortical white matter		MC
Hyperechogenicity thalamus		MC
Hyperechogenicity putamen		MC
PLIC visibility		MC
Four column sign		MC
US after cooling		MC
CT performed		MC
CT normal		MC
Age at first CT	Days	Num
CT report		Free
MRS performed		MC
Date/time of MRS		D/T
Findings		MC
MRI performed		MC
Type of MRI performed		MC
Age at first MRI	Days	Num
Date/time of first MRI		D/T
Sedation used		MC
Sequences performed		MC
Result first MRI		MC
Result first MRI (if abnormal)		Free
Posterior limb of internal capsule		MC
Basal ganglia and thalami		MC
Comment on BGT		Free
Brainstem injury		MC
Cerebellar injury		MC
Diffuse cortical signal abnormality		MC
Parasagittal watershed cortical gray matter injury		MC
White matter		MC
Cortex		MC
Cerebellum		MC
Brain stem		Free
Patterns		MC
Total points Rutherford scale		Num
Gray matter sub score >9.5		MC
Was a second MRI performed		MC
Date/time of MRI 2		D/T
Sedation used?		MC
Result MRI 2		MC
Result MRI 2 (if abnormal)		Free
MRI scoring used	Calgary had separate MRI score worksheet	Num
Weeke score		Num
Other neuroimaging		MC
**Hospital Course/Short-term Outcomes**		
Admitted to NICU		MC
Date/time of admission		D/T
Age at admission (days, minutes)		Num
Weight on admission		Num
Length on admission		Num
Head circumference on admission		Num
SNAP-II score	D1, 2, 3, 4	Num
Temperature on exam		Num
Systemic complications at admission		MC
Any other oral or IV medications		MC
Medication name		Free
Congenital anomalies		MC
If yes, enter code		NUM
If yes, which?		MC
Any congenital disease?		MC
Any metabolic disease?		MC
if yes, enter code		NUM
Any Surgical Procedures?		MC
if yes, enter code		Num
Other adverse events during hospital stay		Free

**NEUROLOGY**		
Neonatal abstinence syndrome		MC
Received sedation during hospitalization		MC
Number of sedation medications reported		Num
Sedation medication		MC
Morphine administered		MC
Maximum dose		Num
Also received for NAS		MC
Congenital neuromuscular disorder present		MC
Describe		Free
Neurological diagnosis		MC
**HEMATOLOGY**		
Any hemotologic diagnosis and/or treatment?		MC
Anemia requiring transfusion?		MC
Hemorrhage requiring immediate transfusion		MC
Thrombocytopenia requiring treatment?		MC
Disseminated intravascular coagulation		MC
Received blood product		MC
Blood product received		MC
Number of RBC transfusions		Num
Date/time of first 10 RBC transfusions		D/T
Date/time of first RBC transfusion		D/T
Date/time of last RBC transfusion		D/T
Age at which RBC transfusion received		Num
Volume of RBC transfusion		Num
Number of platelet transfusions		Num
Date/time of first 10 platelet transfusions		D/T
Date/time of first platelet transfusion		D/T
Date/time of last platelet transfusion		D/T
Age at which platelet transfusion received		Num
Volume of platelet transfusion		Num
Number of FFP transfusions		Num
Date/time of first 5 FFP transfusions		D/T
Date/time of first FFP transfusion		D/T
Date/time of last FFP transfusion		D/T
Age at which FFP transfusion received		Num
Volume of FFP transfusion		Num
Number of cryo transfusions		Num
Date/time of first 5 cryo transfusions		D/T
Date/time of first cryo transfusion		D/T
Date/time of last cryo transfusion		D/T
Volume of cryo administered		Num
Vitamin K administered		MC
Venous thrombus requiring treatment?		MC
Arterial throbosis requiring treatment?		MC
Polycythemia requiring partial exchange transfusion?		MC
**FEEDING/NUTRITION/GROWTH**		
Feeding issues during hospitalization		MC
Nutritional status at admission		MC
Daily weights	D1, 2, 3, 4	Num
Weight		Num
Lenth		Num
Head circumference		Num
Prescribed total fluids	D1, 2, 3, 4	Num
Total daily intake	D1, 2, 3, 4	Num
Total daily intake	ml/kg/day	MC
Calorie intake		Num
Glucose infusion		Num
Type of fluids being administered		MC
Total daily output	D1, 2, 3, 4	Num
Total urine output	ml/kg/hr	MC
Hypoglycemia		MC
Was glucose infusion needed for treatment of hypoglycemia?		MC
Hyperglycemia requiring insulin treatment?		MC
Hyper or hypothyroidism requiring treatment?		MC
Hypocalcemia		MC
Received TPN (parenteral nutrion)		MC

Date/time started TPN		D/T
Age parenteral nutrition started	Hours	Num
Duration of parenteral nutrtion	Hours	Num
Was IV protein given during the 24 hour period?		MC
Amount of protein	gm/kg/day	MC
Are lipids being given?		MC
Amount of lipid	gm/kg/day	MC
Prescribed Na	D1, 2, 3, 4	Num
Was a central line placed	UAC, UVC, PICC, or surgically placed line	MC
UAC placed on admission		MC
UVC placed on admission		MC
Duration central line was in place	Days	Num
Duration of UVC	Hours	Num
Duration of UAC	Hours	Num
Duration of femoral catheter	Hours	Num
Duration of percutaneous CVC	Hours	Num
Duration of nasogastric tube	Hours	Num
Duration of urinary catheter	Hours	Num
Were enteral feeds started		MC
Were enteral feeds started during cooling		MC
Route of enteral feedings		MC
Oral feeding amount	D1, 2, 3, 4	Num
Type of milk during cooling		Free
Feeding type		MC
Feeding (at different time points)	Feedings by mouth include nipple feeding from the breast, nipple feeding from a bottle of human or formula milk, and feedings of human or formula milk by mouth using a feeding cup or spoon.	MC
Date/time first enteral nutrition		D/T
Day of life first enteral feed		Num
How were feeds initially given?		MC
Initial feeding volume		Num
Initial feeding interval		Num
Age continuous enteral feeds started	Hours	Num
Duration of continuous enteral feeds	Hours	Num
Age intermittent enteral feeds started	Hours	Num
Duration of intermittent enteral feeds	Hours	Num
Age oral feeds started	Hours	Num
Duration of oral feeds	Hours	Num
Days to full oral feeds		Num
Initiation of breast feeding	Days	Num
Exclusive breast feedings?		MC
Days of exclusive breast feeding	Days	Num
Age breast feeding started	Hours	Num
Duration of breast feeding	Hours	Num
Probiotics given?		MC
**RESPIRATORY**		
Respiratory rate	BPM	Num
Respiratory rate range		Num
Required respiratory support outside the delivery room		MC
Respiratory support on admission		MC
Mode of CMV on admission		Free
Required respiratory support		MC
Time assisted ventilation initiated		Free
Time assisted ventilation stopped		Free
Required oxygen		MC
Days receiving oxygen		Num
Hood oxygen		MC
Low flow Nasal canula		MC
Start date		D/T
Stop date		D/T
Flow required		MC
Days on nasal cannula		Num
Oxygen saturation range		MC
Hour measurement was taken		Num
Oxygen summary		MC
Oxygenation index	D1, 2, 3, 4	Num
FiO2		Num

Lowest FiO2	0-6 hours; 0-72 hours	Num
Highest fiO2	D1, 2, 3, 4; 0-6 hrs, 0-72 hrs	Num
Days requiring fiO2≥0.21		Num
Days requiring fiO2≥0.4		Num
Surfactant administered	D1, 2, 3, 4	MC
Timing of administration		MC
Mode of ventilation	D1, 2, 3, 4	MC
Days on non-invasive respiratory support		Num
HFNC		MC
Start date		D/T
Stop date		D/T
Days on high flow		Num
CPAP		MC
Start date		D/T
Stop date		D/T
Days on CPAP		Num
Non-invasive respiratory support (NIPPV)		MC
Start date		D/T
Stop date		D/T
Duration of NIPPV		MC
Days on any non-invasive respiratory support	HFNC, NIPPV, CPAP	Num
Required intubation		MC
Date/time intubated		D/T
Date/time extubated		D/T
Assisted ventilation (at different time points)	Answer“Yes” if the infant was given intermittent positive pressure ventilation through an endotracheal tube with a ventilator (conventional or high frequency) at any time on the applicable day of life. Answer “No” if the infant was not given intermittent positive pressure ventilation through an endotracheal tube with a ventilator (conventional or high frequency) at any time on the applicable day of life.	MC
Conventional ventilation used?		MC
Duration of ventilation		MC
Start date		D/T
Stop date		D/T
Days of conventional ventilator		Num
Highest ventilator settings	D1, 2, 3, 4	Free
Highest mean airway pressure (MAP)	D1, 2, 3, 4	Num
Highest rate used		MC
HFOV used?		MC
Start date		D/T
Stop date		D/T
Days of HFOV		Num
HFJV used?		MC
Start date		D/T
Stop date		D/T
Days of HFJV		Num
Days on invasive ventilation		Num
Duration of total respiratory support	Days	Num
Duration of total oxygen support	Days	Num
Required paralytic		MC
Any respiratory complications		Free
Severe pulmonary disease		MC
TTN		MC
RDS	need of surfactant and ventilation or need of ventilation (nasal cannula, high flow nasal cannula, CPAP) over the first days of life	MC
Meconium aspiration syndrome	if meconial amniotic fluid and significant postnatal lung disease	MC
Airway malformation		MC
Pulmonary airleak		MC
Developed during		MC
Pneumothorax		MC
Pneumothorax Evacuated?		MC
Where did the pneumothorax occur?		MC
Pulmonary hemorrhage		MC
Alveolar capillary dysplasia		MC
Pneumatocele		MC

Pulmonary Interstitial Emphysema		MC
BPD with pulmonary hypertension		MC
BPD		MC
Oxygen at 28 days?		MC
Systemic steroid for pulmonary reason?		MC
Dexamethasone used		MC
Start Date		D/T
Stop Date		D/T
Cumulative Dose		Num
Total cumulative Dose		Num
Stimulants used?		MC

CARDIOLOGY

Any Circulatory problem or treatment?		MC
Heart rate	BPM	Num
Highest recorded heart rate	BPM	Num
Heart rate range		MC
Hour the measurement was taken		Num
Systolic BP	mm Hg	Num
Lowest systolic BP	mm Hg	Num
Diastolic BP	mm Hg	Num
Lowest diastolic BP	mm Hg	Num
Was MAP assessed at least once		MC
MAP recorded by		MC
Mean BP	mm Hg	Num
MAP range		MC
Hour the measurement was taken		Num
Lowest mean BP	mm Hg	Num
Was an echo performed		MC
Timing of first echo		D/T
Findings from the echo		MC
Tricuspid regurgitation		MC
Mitral regurgitation		MC
Contractility disorder		MC
Worst Echo	hours	Num
Worst Echo findings		Free
ECG performed		MC
ST depression and T wave inversions		MC
Arrhythmias requiring treatment		MC
Congenital heart disease		MC
Congenital heart disease requiring prostaglandin		MC
Cardiac dysfunction	Evidence of cardiac dysfunction includes myocardial dysfunction and/or tricuspid insufficiency on physical exam, echocardiogram, or cardiac catheterization. Arrhythmias do not count as evidence of cardiac dysfunction for this question.	MC
Required inotropes during NICU stay		MC
When were inotropes started	hours of life	Num
Number of blood pressure medications reported		Num
How long were vasoactive medications required	Hours;days	Num
Dopamine		MC
Dose		Free
Age when initiated		Num
Age when completed		Num
Total duration		Num
Norepinephrine		MC
Dobutamine		MC
Dose		Free
Age when initiated		Num
Age when completed		Num
Total duration		Num
Epinephrine		MC
Dose		Free
Age when initiated		Num
Age when completed		Num
Total duration		Num
Vasopressin		MC
Dose		Free
Hydrocortisone		MC
Start Date		Date

Stop Date		Date
Total Days on Hydrocortisone		Num
Milrinone		MC
Dose		Free
VIS score		Num
Persistent pulmonary hyptertension of newborn	Diagnosis of Persistent Pulmonary Hypertension of the Newborn (PPHN), occurring at any time prior to discharge from your hospital, was documented in the infant medical record	MC
Required iNO		MC
Date/time started on iNO		D/T
Date/time stopped iNO		D/T
Days on iNO		Num
Required ECMO		MC
What type		MC
Duration		Num
PDA?		MC
PDA medical treatment		MC
PDA Ligation?		MC
**RENAL/FLUIDS**		
Received crystalloid for volume expansion in the first 12 hours		MC
NaCl bolus needed		MC
Number of NaCl boluses		Num
Received bolus of buffering medication in first 12 hours	Does not include if only added to continuous IV fluids or TPN	MC
Albumin 5%		MC
Albumin 25%		MC
Renal injury/failure	Acute or chronic	MC
Need for replacement therapy		Free
Peritoneal dialysis		MC
Hemodialysis		MC
Renal ultrasound performed?		MC
Increased echogenicity		MC
Acute tubular necrosis		MC
Proteinuria (≥2+)		MC
Age at first void		Num
Diuresis	ml/kg/hr	Num
Oliguria		MC
**GI/HEPATIC**		
Any Gastrointestinal diagnosis requiring treatment?		MC
Hepatic dysfunction	If serum AST (SGOT) and/or ALT (SGPT) were obtained prior to discharge from your hospital and one or both values were elevated above local normal laboratory values.	MC
Persistent bloody gastric residuals and/or recurrent vomiting		MC
Bloody stool and/or HI bleeding with hct drop ≥2 g/dL		MC
NEC >/+2		MC
If yes, which category		MC
Laparotomy done for NEC?		MC
Focal intestinal performation?		MC
Cholestasis?		MC
Hyperbilirubinemia within 7 days of birth	if a serum indirect bilirubin level ≥ 20 mg/dl (342 micromoles/L) was recorded in the infant medical record for a specimen obtained at any time within 7 days of birth.	MC
Hyperbilirubinemia requiring treatment		MC
**INFECTIOUS**		
Any infectios problem or treatment?		MC
Organism identified		MC
Type of organism		MC
Source of culture		Free
Infection/sepsis		MC
Timing		MC
If yes, pathogen		Free
Early onset sepsis/meningitis < 72 hours?		MC
If yes, pathogen:	Code for pathogen	Num
EOS day of life		Num

Where was the early onset sepsis/meningitis diagnosed?		MC
Clinical early onset sepsis/infection with negative blood work <72hours?		MC
Proven late onset/nosocomial sepsis/meningitis?		MC
Episode 1 pathogen	Code	Num
Episode 1 sepsis/meningitis		MC
Episode 1 Lineinfection		MC
Episode 1 occured at		MC
Episode 2 pathogen	Code	Num
Episode 2 sepsis/meningitis		MC
Episode 2 Lineinfection		MC
Episode 2 occured at		MC
Episode 3 pathogen	Code	Num
Episode 3 sepsis/meningitis		MC
Episode 3 Lineinfection		MC
Episode 3 occured at		MC
Episode 4 pathogen	Code	Num
Episode 4 sepsis/meningitis		MC
Episode 4 Lineinfection		MC
Episode 4 occured at		MC
Blood culture obtained		MC
Was pathogenic bacteria identified		MC
What bacteria		Free
Urine culture obtained		MC
Was pathogenic bacteria identified		MC
What bacteria		Free
CSF meningitis Film Array panel results		Free
CSF culture obtained		MC
Was pathogenic bacteria identified		MC
Meningitis DOL		Num
What bacteria		Free
Bacterial meningitis or encephalitis	Infant was suspected or proven to have bacterial meningitis or encephalitis at any time prior to discharge from your hospital, as recorded in the infant medical record. Suspected meningitis or encephalitis includes cases in which no organism was recovered from the CSF or brain biopsy but clinical findings, serology, and/or CSF findings suggest meningitis or encephalitis. Answer“Proven” if the infant had bacterial meningitis or encephalitis documented in the medical record at any time prior to discharge from your hospital, with a positive culture of the CSF or brain biopsy specimen.	MC
Type of organism		MC
Onset of meningitis or encephalitis	Early = within 3 days of birth; Late = more than 3 days after birth	Num
Fungal meningitis or encephalitis	Infant was suspected or proven to have fungal meningitis or encephalitis at any time prior to discharge from your hospital, as recorded in the infant medical record. Suspected meningitis or encephalitis includes cases in which no organism was recovered from the CSF or brain biopsy but clinical findings, serology, and/or CSF findings suggest meningitis or encephalitis. Answer“Proven” if the infant had fungal meningitis or encephalitis documented in the medical record at any time prior to discharge from your hospital, with a positive culture of the CSF or brain biopsy specimen.	MC
Onset of meningitis or encephalitis	Early = within 3 days of birth; Late = more than 3 days after birth	Num

Viral meningitis or encephalitis	Infant was suspected or proven to have viral meningitis or encephalitis at any time prior to discharge from your hospital, as recorded in the infant medical record. Suspected meningitis or encephalitis includes cases in which no organism was recovered from the CSF or brain biopsy but clinical findings, serology, and/or CSF findings suggest meningitis or encephalitis. Answer"Proven" if the infant had viral meningitis or encephalitis documented in the medical record at any time prior to discharge from your hospital, with a positive culture of the CSF or brain biopsy specimen.	MC
Onset of meningitis or encephalitis	Early = within 3 days of birth; Late = more than 3 days after birth	Num
HSV culture or PCR obtained		MC
Was it positive		MC
Any congenital infections?		MC
If yes, pathogen	Code for pathogen	Num
Pneumonia		MC
Type of pneumonia		MC
Bronchiolitis?		MC
Antibiotics		MC
Any Antibiotics given <72 hours of life?		MC
Total number of antibiotics		Num
Antibiotic name		MC
List antibiotics		Free
Duration of antibiotics	Days	Num
Antiviral name		MC
Antifungal name		MC
**DISCHARGE**		
Hearing screen (ABR) normal?		MC
if ABR abnormal		MC
Length of hospital stay	Days	Num
Length of stay in NICU	Days	Num
Feeding issues at discharge		MC
Feeds at DC (if issues present)		MC
Feeding at discharge	Feedings by mouth include nipple feeding from the breast, nipple feeding from a bottle of human or formula milk, and feedings of human or formula milk by mouth using a feeding cup or spoon.	MC
Type of feeding at discharge		MC
Type of milk on discharge		Free
Mothers breast milk at discharge?	Partial or all	MC
Discharged on TPN?		MC
Discharged on oxygen?		MC
Discharged on Monitor?		MC
Tracheostomy at discharge		MC
Discharged with ventilator		MC
Discharge medications		MC
Anticonvulsants at discharge		MC
Phenobarbital		MC
Phenytoin or fosphenytoin		MC
Lorazepam		MC
Diazepam		MC
Midazolam		MC
Benzodiazepine		MC
Levetiracetam		MC
Topiramate		MC
Other anticonvulsants		MC
Medical equipment at discharge		MC
List all diagnoses at discharge		Free
Neuro exam normal at discharge		MC
Clinical neurological exam at discharge		MC
Details of abnormal neuro exam		Free
Hammersmith Neurological Exam at discharge	Score	Num
GMA at discharge		MC
Altered muscle tone at discharge		MC
Reflexes at discharge		MC

General movements at discharge		MC
Behavior at discharge		MC
Visual and auditory orientation at discharge		MC
Length at discharge	cm	Num
Head circumference at discharge	cm	Num
Weight at discharge		Num
Status at discharge		MC
Date of discharge		D/T
Date of transfer		D/T
Discharged with hospice care		MC
Date of death		D/T
Time of death	Hours of life	Num
Primary cause of death?		MC
Location of death		MC
Organ or tissue donor?		MC
Autopsy status		MC
Did death occur < 12 hours after admission to the NICU?		MC
Was intesive care withdrawn or limited prior to death		MC
If support withdrawn, criteria for withdrawal		Free
If support not withdrawn, cause of death		Free
Information in medical record about end of life decisions		MC
Clear prognostiy information in the record		MC
Decision-making process involved parents		MC
Were all medications withdrawn except for sedation/analgesia		MC
Were parents offered specialized psychological support		MC
Were parents offered religious support		MC
Baby died in parents' arms		MC
There were other family members accompanying parents		MC
Is there a continued presence of the physician during palliative care		MC
Were interviews arranged to examine the mourning process		MC
Number of interviews with the family in EoL decision process		Num
Benzos used prior to EoL decision		MC
Benzos used after EoL decision		MC
Fentanyl/morphine used prior to EoL decision		MC
Fentanyl/morphine used after EoL decision		MC
Phenobarbital used prior to EoL decision		MC
Phenobarbital used after EoL decision		MC
Drug doses increased?		MC
Information in clinical record on discomfort		MC
Timing of process noted		MC
Hours to death from start of EoL decision		Num
Follow up planned?		MC
Referrals and/or appointments at discharge		MC
Month of life follow up planned		Free
Was the infant referred for outpatient neurology follow up		MC

Follow up

No timing specified

Bayley testing performed?		MC
Bayley		Num
Age of testing		Num
Cognitive score		Num
Language score		Num
Motor score		Num
Second Bayley testing performed?		MC
Age of testing		Num
Cognitive score		Num
Language score		Num
Motor score		Num
CP		MC
Type		MC
Severity		MC
Epilepsy		MC
Global developmental delay		MC
Hearing loss		MC
Speech delay		MC
Microcephaly		MC
Follow-up with		MC
Follow-up date		D/T
Follow-up type/location		MC
Follow-up age		Num

Follow up missed	MC
Reason for missed follow up	MC
<u>Prior to 49 weeks PMA</u>	
GMA	MC
<u>49-60 weeks PMA</u>	
Fidgety movements	MC
<u>6-10 mo</u>	
Length	Num
Weight	Num
Head circumference	Num
Survey of Wellbeing of Young Children (SWYC) Milestone	MC
SWYC Baby Pediatric Symptom Checklist	MC
SWYC Cognitive Fusion Questionnaire	MC
Edinburgh Maternal Depression Screening	Num
Referred to school services	MC
Qualified for school services	MC
Refused school services	MC
Received PT through school	MC
Received OT through school	MC
Received speech therapy through school	MC
Ages & Stages Questionnaire	MC
Bayley Infant Neurodevelopmental Screener (BINS) Cognitive	MC
BINS Receptive	MC
BINS Expressive	MC
BINS Fine Motor	MC
BINS Gross Motor	MC
BSID Cognitive	Num
BSID Language	Num
<u>16-20 mo</u>	
Length	Num
Weight	Num
Head circumference	Num
Referred to school services	MC
Qualified for school services	MC
Refused school services	MC
Received PT through school	MC
Received OT through school	MC
Received speech therapy through school	MC
Ages & Stages Questionnaire	MC
Communication and Symbolic Behavior Scales	MC
Bayley Infant Neurodevelopmental Screener (BINS) Cognitive	MC
BINS Receptive	MC
BINS Expressive	MC
BINS Fine Motor	MC
BINS Gross Motor	MC
MCHAT-R	MC
<u>2 years</u>	
Weight	Num
OFC	Num
Microcephaly	MC
Length	Num
Illnesses/hospitalization	Free
Referred to school services	MC
Qualified for school services	MC
Refused school services	MC
Received PT through school	MC
Received OT through school	MC
Received speech therapy through school	MC
Ages & Stages Questionnaire	MC
Communication and Symbolic Behavior Scales	MC
BSID Global Motor	Num
BSID Gross Motor	Num
BSID Fine Motor	Num
BSID Cognitive	Num
BSID Language	Num
BSID Receptive Language	Num

BSID Expressive Language	Num
BSID Socio-emotional	Num
MCHAT-R	MC
Altered muscle tone	MC
Persistence of primitive reflexes	
Increased myotactic reflexes	
Epilepsy	
Age of onset	
Recurring	
Partial seizures	MC
Febrile seizures	MC
Generalized seizures	MC
Seizure control	MC
Number of seizure medications	Num
Sensorineural hearing loss	MC
Other neurological deficits	Free
Drugs	Free
Cerebral palsy	MC
Spastic	MC
Dyskinetic	MC
Distribution of spasticity	MC
GMFCS	MC
Manual ability classification system	MC
CBCL Syndrome Scale Score	Num
CBCL DSM IV Oriented Scale Score	Num
3 years	
Length	Num
Weight	Num
Head circumference	Num
Referred to school services	MC
Qualified for school services	MC
Refused school services	MC
Received PT through school	MC
Received OT through school	MC
Received speech therapy through school	MC
Ages & Stages Questionnaire	MC
