# Supplementary material for: Data Collection Variability Across Neonatal Hypoxic-Ischemic Encephalopathy Registries
Source: J Pediatr. Author manuscript; Available in PMC 2026 Jul 21. (PMC13384812; doi:10.1016/j.jpeds.2025.114476)
Supplement: Supp 5 [file NIHMS2186118-supplement-Supp_5.docx]

**Data Statement:** All of the data supporting the findings of this study can be found within the article or its supplementary materials.
