# Supplementary material for: Data Collection Variability Across Neonatal Hypoxic-Ischemic Encephalopathy Registries
Source: J Pediatr. Author manuscript; Available in PMC 2026 Jul 21. (PMC13384812; doi:10.1016/j.jpeds.2025.114476)
Supplement: Supplemental Table 2 [file NIHMS2186118-supplement-Supplemental_Table_2.pdf]

Response from:	What term is used in your registry to define the population?	Please describe which term is used	What definition is used to define whichever term you selected in the question above?	What are the inclusion criteria for your registry? (choice=All those treated with therapeutic hypothermia)	What are the inclusion criteria for your registry? (choice=All those referred for potential therapeutic hypothermia)	What are the inclusion criteria for your registry? (choice=Other)	Provide detail of inclusion criteria used	Are there specific exclusion criteria for your registry?	What are the exclusion criteria?
Registry Contact	Hypoxic-ischemic encephalopathy		Moderate to severe HIE eligible for TH	Unchecked	Unchecked	Checked	All eligible for TH	Yes	Same as exclusion criteria for TH (GA<35, BW<1800, major congenital anomalies)
Registry Contact	Hypoxic-ischemic encephalopathy		1. Documented evidence of an acute perinatal event such as fetal distress, cord prolapse, uterine rupture, reduced fetal movements, abruption, antepartum hemorrhage or emergency cesarean section due to fetal distress 2. Evidence of intrapartum hypoxia: at least one of: Apgar score of ≤5 at 10 minutes; Mechanical ventilation or resuscitation within 10 minutes; Cord pH < 7.00 (venous or arterial), or an infant arterial pH < 7.00 or base deficit ≥ 12 within 60 minutes of birth	Unchecked	Unchecked	Checked	all those admitted to canadian tertiary sites that enter all eligible data in CNN + with a diagnosis of HIE (includes non cooling and cooling babies) Note: some sites dont enter data on HIE (approx 3 sites) and other sites (2) only enter data on HIE + cooling babies (rest of sites enter the data for any NICU admission with HIE as described above)	Yes	babies in Level 1-2 NICUs, babies that died in Level 1-2 units, stillbirths
Registry Contact	Hypoxic-ischemic encephalopathy		All 3: 1. Presence of a clinically recognized encephalopathy within 72 hours of birth. Encephalopathy is defined as the presence of 3 or more of the following within 72 hours of birth: a. Abnormal level of consciousness: hyperalertness, lethargy, stupor or coma b. Abnormal muscle tone: hypertonia, hypotonia or flaccidity c. Abnormal deep tendon reflexes: increased, depressed, or absent d. Seizures: subtle, multifocal or focal clonic e. Abnormal Moro: exaggerated, incomplete, or absent f. Abnormal suck: weak or absent g. Abnormal respiratory pattern: periodic, ataxic, or apnoeic h. Oculomotor or papillary abnormalities: skew deviation, absent or reduced Doll's eye or fixed unreactive AND 2. Three or more supporting findings from the following list: a. Arterial cord pH b. Apgar score at 5 minutes of 5 or less c. Evidence of multi-organ system dysfunction within 72 hours of birth d. Evidence of foetal distress on antepartum monitoring: persistent late decelerations, reversal of end-diastolic flow on Doppler of the umbilical artery, or BPP of 2 or less e. Evidence of CT, MRI, technetium, or US brain scan performed within 7 days of birth of diffuse or multifocal ischemia or cerebral edema f. Abnormal EEG: low amplitude and frequency, periodic, paroxysmal or isoelectric AND 3. The absence of an infectious cause, a congenital malformation of the brain or an inborn error of metabolism which could explain encephalopathy	Unchecked	Unchecked	Checked	All HIE neonates admitted to participating NICUs	Yes	Death prior to admission
Registry Contact	Neonatal encephalopathy		Any infant receiving hypothermia for neonatal encephalopathy (presumed to by HIE but we did not require sites to exclude if other non-HIE etiologies found in cooled infants)	Unchecked	Unchecked	Checked	All those who were cooled or received PPV in delivery room and either 1) had cord blood gas pH≤7.1 or 2) had no cord blood gas obtained.	Yes	Cyanotic heart disease, known fetal CNS anomalies, wards of the state
Registry Contact	Asphyxia		All neonates referred to the NICU for potential cooling	Unchecked	Checked	Unchecked		No	
Registry Contact	Hypoxic-ischemic encephalopathy		Infants are considered to have HIE if they met the following three criteria: 1) at least one of the following clinical surrogates of hypoxic ischemic insult: altered fetal heart rate pattern (non-reassuring fetal status), sentinel event, or labor dystocia; 2) Apgar score <5 at 5 and 10 minutes, need for resuscitation, including mask ventilation for more than 10 minutes after birth or endotracheal intubation, or acidosis (pH <7.0 and/or base deficit >16 mmol/L) in umbilical cord blood or within 60 minutes from birth; and 3) early neonatal encephalopathy defined as a syndrome of neurological dysfunction in the first 6 hours manifested by a subnormal level of consciousness with or without seizures (moderate or severe HIE) or palmary hyperexcitability, tremor, overactive myotatic reflexes, hypersensitivity to stimulation, or startle responses (mild HIE).	Checked	Checked	Checked	We include in the registry infants with any degree of severity of HIE (Mild, moderate or severe) But we only cooled infants with moderate and severe EHI. We used whole-body cooling (Techotherm TSmed 200 N or Criticool, MTRE Ltd.).	Yes	a)To have congenital abnormalities. b)Presented neonatal collapse. c)To have other identifiable etiologies of neurological dysfunction such as infection or genetic disease. d)Parental consent not granted.
Registry Contact	Other	Therapeutic hypothermia	We include any baby treated with hypothermia	Checked	Unchecked	Unchecked		No	
Registry Contact	Hypoxic-ischemic encephalopathy		based on thompson7Sranat score and clinical history (A and B critera)	Unchecked	Unchecked	Checked	cooled or not cooled more recently also all infants with	Yes	to treatment, severe congenital malformation such for
Registry Contact	Hypoxic-ischemic encephalopathy		Either: - Apgar 10min =/<5 - Ongoing resuscitation including resp support CPAP or higher at 10 min - pH <7.0 (cord or baby gas within 1 hour) - BD > 12 (cord or baby gas within 1 hour) AND Sarnat exam consistent with moderate or worse encephalopathy between 1-6 hours of life	Unchecked	Unchecked	Checked	We include all babies and HIE is part of it with a more detailed data entry	No	
Registry Contact	Hypoxic-ischemic encephalopathy		Definition as in Toby Trial - Criteria A, B, C	Checked	Unchecked	Unchecked		Yes	Prematurity (<36+0) Congenital malformations Birth weight less 1800g Intracerebral hemorrhage or thrombosis
Registry Contact	Hypoxic-ischemic encephalopathy		NICHD criteria for HIE definition and TH	Checked	Unchecked	Unchecked		No	
Registry Contact	Hypoxic-ischemic encephalopathy		Infants with Neonatal Encephalopathy due to a possible hypoxic ischemic event either diagnosed clinically or by MRI	Unchecked	Unchecked	Checked	Infants treated with Hypothermia Infants diagnosed with neonatal encephalopathy due to hypoxic ischemic event	Yes	Main cause of encephalopathy not hypoxic ischemic Less than 34 weeks gestation
Registry Contact	Hypoxic-ischemic encephalopathy		Infants who met the following criteria and were born at or transferred to a participating center at ≥34 weeks GA with evidence of HIE were eligible for enrollment in the registry: 1. One or more of the following: i. Apgar score <5 at 5 min, ii. Metabolic acidosis [Base deficit (BD) > -16 mmol/L at cord blood gas or blood gas analysis at 1st h after birth), iii. Delayed onset of respiration for five or more minutes, iv. Birth via emergency cesarean section due to fetal distress 2. Need for ventilation immediately after birth (positive pressure ventilation or intubation) 3. Evidence of encephalopathy (lethargy, hypotonia, altered state of consciousness, weakness/absent of reflexes and/or seizures) 4. Multiorgan involvement [encephalopathy and at least one other organ system other than central nervous system (CNS)].	Unchecked	Checked	Unchecked		No	
Publication	Asphyxia		Infants >1500 grams and at least one of the following: 1) admitted with suspected encephalopathy or suspected perinatal asphyxia; 2) 5-min Apgar <=3 or 10 min Apgar <=4; 3) received active hypothermia; 4) diagnosis with HIE	Unchecked	Unchecked	Checked	Any infant born into a network hospital	Yes	Delivery room death
Publication	Neonatal encephalopathy		hyperalertness, irritability, lethargy or obtundation, coma, decreased spontaneous activity, hypotonicity or flaccidity, decerebrate posturing, absent or weak suck, abnormal pupillary reflex, abnormal Moro reflex, persistent bradycardia, periodic breathing or apnea	Unchecked	Checked	Checked	Neonates >=34 weeks GA and concern for encephalopathy or seizure and any one of the following: 1) sentinel event prior to delivery such as uterine rupture, profound bradycardia, or cord prolapse; 2) low Apgar <=5 at 10 min; prolonged resuscitation>10 min; pH<7.1 from cord or patient within 60 min; BD<-10 mEq/L from cord or patient within 60 min	No	
Publication	Neonatal encephalopathy		presence of seizures and/or altered consciousness (stupor, coma)	Checked	Unchecked	Checked	Born >=36 weeks gestation with NE within 3 days of birth, 5 min Apgar score <=3, infants receiving neuromuscular blockade, and any infant receiving HT	Yes	Born with CNS birth defect
Publication	Hypoxic-ischemic encephalopathy		Encephalopathy within 72 hours of birth (3 or more of 8 neuro findings) AND absence of infectious cause, congenital brain malrofmation of the brain or inborn error of metabolism AND 3 or more of: arterial cord pH<7.0; Apgar score at 5 min <=5; evidence of multi-organ dysfunction; evidence of fetal distress on antepartum monitoring; evidence of diffuse multifocal ischemia or cerebral edema within 7 days of birth; or abnormal EEG	Unchecked	Unchecked	Checked	Any infant admitted to a network hospital	No	
Publication	Other	Therapeutic hypothermia		Checked	Unchecked	Unchecked		Yes	Not cooled; no moderate/severe encephalopathy; GA<36 weeks; age>6h
Publication	Other	Therapeutic hypothermia		Checked	Unchecked	Unchecked		No	
